# Ycf93 (Orf105), a Small Apicoplast-Encoded Membrane Protein in the Relict Plastid of the Malaria Parasite *Plasmodium falciparum* That Is Conserved in Apicomplexa

**DOI:** 10.1371/journal.pone.0091178

**Published:** 2014-04-04

**Authors:** Christopher D. Goodman, Geoffrey I. McFadden

**Affiliations:** School of Botany, University of Melbourne, Parkville, Victoria, Australia; Institut national de la santé et de la recherche médicale - Institut Cochin, France

## Abstract

Malaria parasites retain a relict plastid (apicoplast) from a photosynthetic ancestor shared with dinoflagellate algae. The apicoplast is a useful drug target; blocking housekeeping pathways such as genome replication and translation in the organelle kills parasites and protects against malaria. The apicoplast of *Plasmodium falciparum* encodes 30 proteins and a suite of rRNAs and tRNAs that facilitate their expression. *orf*105 is a hypothetical apicoplast gene that would encode a small protein (*Pf*Orf105) with a predicted C-terminal transmembrane domain. We produced antisera to a predicted peptide within *Pf*Orf105. Western blot analysis confirmed expression of *orf*105 and immunofluorescence localised the gene product to the apicoplast. *Pforf*105 encodes a membrane protein that has an apparent mass of 17.5 kDa and undergoes substantial turnover during the 48-hour asexual life cycle of the parasite in blood stages. The effect of actinonin, an antimalarial with a putative impact on post-translational modification of apicoplast proteins like *Pf*Orf105, was examined. Unlike other drugs perturbing apicoplast housekeeping that induce delayed death, actinonin kills parasites immediately and has an identical drug exposure phenotype to the isopentenyl diphosphate synthesis blocker fosmidomycin. Open reading frames of similar size to *Pf*Orf105, which also have predicted C-terminal trans membrane domains, occur in syntenic positions in all sequenced apicoplast genomes from Phylum Apicomplexa. We therefore propose to name these genes *ycf*93 (hypothetical chloroplast reading frame 93) according to plastid gene nomenclature convention for conserved proteins of unknown function.

## Introduction

The widely used malaria prophylactic, doxycycline, was originally developed as an antibacterial that blocks protein translation by prokaryotic ribosomes [1]. Efficacy against malaria is believed to result from inhibition of translation in the relict plastids (apicoplast) of malaria parasites [2]. Other inhibitors of prokaryotic translation, plus inhibitors of apicoplast DNA replication (fluoroquinolones like ciprofloxacin) are also paraciticidal [2], [3]. Indeed, any perturbation of apicoplast housekeeping appears to kill malaria parasites [2], [3]. This highlights the importance of the maintenance of the apicoplast for parasite survival.

Apicoplasts arose by secondary endosymbiosis of a red alga within the common ancestor of Apicomplexa and dinoflagellates [4]. Most dinoflagellates remain photosynthetic, but Apicomplexa adopted a parasitic lifestyle and dispensed with photosynthesis [5], [6]. The endosymbiotic organelle, however, persists and is indispensable [7]. The apicoplast of the human malaria parasite *Plasmodium falciparum* has a small (35 kb), circular genome similar to that of most plastids; the major difference being that the apicoplast genome encodes no genes for photosynthesis [8]. Thirty putative protein-encoding genes have been identified on the *P. falciparum* apicoplast genome, and seven of these are hypothetical proteins (open reading frames >50 amino acids) [8]. With the exception of SufB/ORF470, which has a role in the formation of iron∶sulphur clusters, and ClpC, a protein chaperone subunit with a possible role in protein import [9], all apicoplast genes with known roles are devoted to expression of the genome (housekeeping) [8]. Further information of the roles of these genes, particularly the hypothetical ones, could thus be valuable in targeting apicoplasts in the battle against malaria.

To explore the roles of hypothetical apicoplast proteins we attempted to generate antisera to predicted peptides from a selection of the hypothetical apicoplast genes (ORFs) that possibly encode proteins. Here we describe the characterization of one previously hypothetical apicoplast protein – Orf105/*Pf*Ycf93 - to which we generated a successful antiserum allowing us to localize the protein to the apicoplast membranes, document its turnover during the cell cycle, and explore effects of a post-translation inhibitor on the apicoplast and *Pf*Ycf93. We also searched for homologues of *Pfycf93* in the apicoplast genomes of other apicomplexan parasites and related photosynthetic symbionts of corals such as the chromerids *Chromera velia* and *Vitrella brassicaformis*.

## Results and Discussion

### *Pf*Ycf93 is an apicoplast-encoded protein

*orf*105 is an open reading frame (366 nucleotides) located on the IR-B section of the 35 kb circular genome of the apicoplast of *P. falciparum* (GenBank: X95276.2) that encodes a hypothetical protein of 121 amino acids [8]. The name *orf*105 stems from a previous annotation for a shorter version of the protein that invokes a downstream Met based on the presence of a transcribed and processed *trnS* gene within the N-terminus of the 121 amino acid open reading frame [10]. In keeping with plastid gene nomenclature convention for gene products conserved in multiple species [11] we designated the gene *ycf93* (hypothetical chloroplast open reading frame 88) because orthologues occur in numerous Apicomplexa (see below).

**Figure 1:**
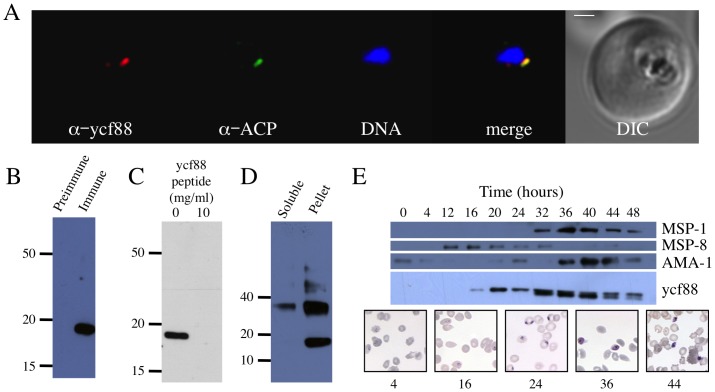
Characterization of *Pfycf93* gene product in *Plasmodium falciparum*. A) Localization in the apicoplast of an antigen recognised by *PfYcf93* peptide antisera. *Pf*Ycf93 (red) localises to a small punctum that overlaps with the apicoplast marker acyl carrier protein (green) and sits beside the nucleus (blue) in a small, ring-stage parasite within a red blood cell. Bar = 1 µm. B) Western blot of SDS-PAGE separated proteins labelled with either pre-immune or immune sera demonstrates specific labelling of a 17.5 kDa protein. C) Labelling of the 17.5 kDa protein with the peptide antiserum is abrogated by pre-incubation of the membrane with 10 mg/ml of peptide. D) *Pf*Ycf93 protein partitions into the pellet during Triton X-114 partitioning, and a second band of 35 kDa is apparent. E) Western blotting of the 17.5 kDa band across the 48 hour asexual red blood cell cycle of synchronized parasites demonstrates that *Pf*ycf93 is not detectable at 0, 4, or 12 hours but begins to be detectable at 16 hours and then increases in abundance from 20 to 40 hours and then decreases again at 44 and 48 hours. The change in abundance of the 17.5 kDa band is similar to published fluctuations of merozoite surface protein 1 (MSP-1), but contrasts to MSP-8 and apical merozoite antigen 1 (AMA-1), which have very different turnover patterns. Giemsa stained images of parasite development at different time points are shown. Loading was normalised by protein amount and equal loading confirmed by Ponceau staining (data not shown).

Goat antisera raised against a 14-residue peptide (NILLKSKNSNNYIY) were used for immunofluorescence on *in vitro* cultured blood stage *P. falciparum* parasites (Fig. 1A). Antigen (red) was localized to a small punctum (Fig. 1A). Co staining (green) with antisera to the apicoplast marker acyl carrier protein (ACP) [12] showed the antigen recognised by our goat antiserum co localized with the apicoplast adjacent to the nucleus (Fig. 1A). We conclude that the peptide antisera recognises an antigen located in the apicoplast.

### *Pf*Ycf93 is an apicoplast membrane protein and may form dimers

Western blotting using the peptide antisera identified a single band of 17.5 kDa (Fig. 1B), which is in reasonable agreement with the predicted mass for *Pf*Orf105/*Pf*Ycf93 of 15 kDa. Controls with pre-immune sera did not decorate any bands (Fig. 1B). Specificity of the antisera for *Pf*Ycf93 was verified by pre-incubation of the antiserum with 10 mg/ml peptide, which abrogated binding to the 17.5 kDa band (Fig. 1C). We conclude that the antiserum is specific for the gene product of the apicoplast gene *Pforf*105, confirming it as a protein-encoding gene, which we rename *Pfycf93*.

*Pf*Ycf93 protein partitions into the detergent phase during Triton X-114 phase partitioning [13], indicating that it is a membrane protein (Fig. 1D). This membrane localization confirms the bioinformatic predictions of a single C-terminal transmembrane domain. In detergent partitioned non-denaturing Western blots, a second major band of apparent molecular mass 37 kDa was observed (Fig. 1D), indicating that *Pf*Ycf93 may exist as a dimer. Based on the immunofluouresence, Western blots, and detergent partitioning we conclude that *Pfycf93* encodes a small apicoplast membrane protein. Considering the apicoplast appears to lack any systems such as YidC/Oxa/Alb3 chaperone family proteins involved in insertion, export or assembly of membrane proteins [14], [15], it seems most likely that *Pf*Ycf93 is located in the innermost of the four bounding membranes and self inserts.

### *Pf*Ycf93 undergoes turnover

**Figure 2:**
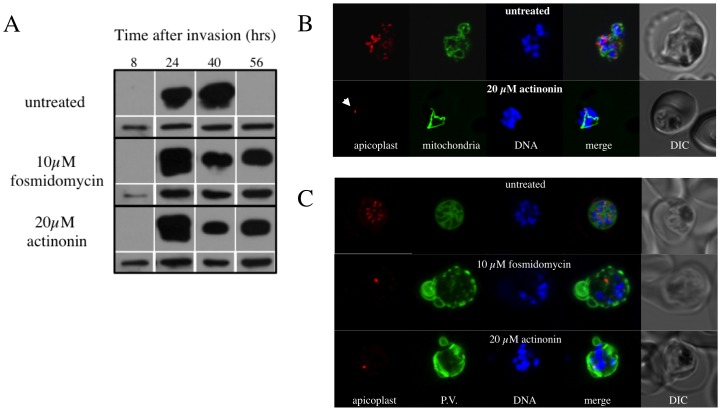
The effects of the prokaryotic post-translational modification inhibiting drug actinonin on *Pf*Ycf93 turnover and apicoplast development and cytokinesis. A) Western blot showing the effect of actinonin and the isoprenoid precursor synthesis inhibitor fosmidomycin on steady state levels of *Pf*Ycf93 over one life cycle. Treatment with these drugs abrogates the normal turnover of *Pf*Ycf93 observed during schizont (40 hour) to ring (56 hour) transition. Loading controls at each life cycle stage using anti-BiP are shown in the lower panels. B) Effect of the putative post-translational inhibitor actinonin on biogenesis of the apicoplast, mitochondria and nuclei. Live cell imaging of untreated parasite with red fluorescent apicoplast, green fluorescent mitochondrion and blue fluorescent nuclei shows the normal branching/division of the apicoplast, elaboration of the mitochondrion and multiplication of the nucleus at the schizont stage. Actinonin-treated parasite at the same stage shows a branched mitochondrion and multiple nuclei but an undeveloped, stunted apicoplast (arrow). C) Effect of fosmidomycin and actinonin on the apicoplast and parasitophorous vacuole (P.V.) at the schizont stage. Immunofluorescence assay of untreated parasite with red fluorescent apicoplast (anti-ACP), green P.V. and blue fluorescent nuclei shows the segregation of the apicoplast, nuclei into distinct merozoites during late schizont stage. Actinonin or fosmidomycin treated parasites at the same stage show multiple nuclei but an undeveloped apicoplast and severe disruption of internal membrane structure in the parasite.

During the 48-hour life cycle of malaria parasites the apicoplast undergoes dramatic growth, branching, and division resulting in the distribution of an apicoplast into each daughter merozoite [16]. We monitored the steady state levels of *Pf*Ycf93 protein across the life cycle by Western blot on a synchronous culture of *P. falciparum* (Fig. 1E & 2A). In the initial part of the life cycle just following invasion (0 hours), the so-called ring stage parasites did not have any detectable *Pf*Ycf93 (Fig. 1E). After 4 hours (early rings) and 12 hours (early trophozoites) *Pf*Ycf93 was still not detectable (Fig. 1E). At 16 hours a faint *Pf*Ycf93 band had developed, and by 20 hours (late trophozoites) there was a robust *Pf*Ycf93 band, which persisted right through the trophozoite phase and into schizogony but then decreased at 44 hours and still further at 48 hours (Fig. 1E). By comparison, probing of the same samples with antibodies to the merozoite surface protein 1 (MSP1) confirms previous reports that MSP1 only begins to be expressed at 32 hours and persists through to early rings (0 hours and 4 hours). Conversely, merozoite surface protein 8 (MSP8) shows a restricted window of expression between 4 and 24 hours, as previously described [17], and the apical merozoite antigen (AMA1) has maximal expression in the shizont/merozoite phase (36 – 0 hours) as described [18].

The drastic disappearance of *Pf*Ycf93 protein toward the latter part of schizogony (Fig. 1E & 2A) suggests that *Pf*Ycf93 undergoes degradation, probably during the late stage of formation of merozoites. This is the first clear example of protein turnover within apicoplasts. Plastids of plants undergo substantial protein turnover due to activity of a range of proteases, most of which are inherited from the cyanobacterial endosymbiotic ancestor of plastids [19]. Three apicoplast proteases (signal processing peptidase [SPP] [20], falcilysin/PreP [21], and caseinolytic protease (Clp) [9], [22], [23]) have been identified in *Plasmodium*. SPP and PreP are believed to have roles in processing of leader peptides that mediate import of nucleus-encoded proteins into the apicoplast [20], [21], so Clp is the prime candidate for turnover of *Pf*Ycf93.

### Is *Pf*Ycf93 subject to post-translational modification?

Like bacteria, plastids use a formyl methionine as the initiator Met for proteins encoded by the plastid genome and synthesised within the plastid [24]. The formyl group is added to the initiator methionine by formyl methionine transferase (FMT) and is later removed from the methionine by peptide deformylase (PDF) after translation [24]. In select instances (depending on the identity of the second amino acid) the deformylated methionine itself is then removed by methionine aminopeptidase (MAP) [24], which influences protein stability [25]. Genes for likely apicoplast FMT, PDF and MAP have been identified in *Plasmodium falciparum* [14], [26] and *Pf*PDF is indeed targeted to the apicoplast [27] suggesting homologous N-terminal processing occurs in the apicoplast. Several drugs inhibit PDF [28]–[32] and one, actinonin, has been noted to have antimalarial activity [33]. Recombinant *Pf*PDF has been expressed and its structure determined [34]. Activity of recombinant *Pf*PDF is inhibited by actinonin in *in vitro* assays [34]. Four apicoplast-encoded proteins from *P. falciparum* (Rps3, Rpl16, Orf91 and Orf105/Ycf93) contain the correct amino acid at residue two [8] to potentially undergo removal of the N-terminal methionine after deformylation by *Pf*PDF. We therefore decided to explore the effect of actinonin on parasite growth, the N-terminus of *Pf*Ycf93, and the turnover of *Pf*Ycf93 during the asexual red blood cell cycle.

*In vitro* culture drug trials of *P. falciparum* 3D7 confirmed reports [33] that the PDF inhibitor actinonin is parasiticidal with an IC_50_ of 4.9 (±1.05) µm for a 48 hour growth assay. Drugs targeting apicoplast housekeeping activities (DNA replication, RNA transcription, and protein translation) typically result in the curious phenomenon known as delayed death in apicomplexan parasites [3], [7]. Delayed death alludes to the fact that the drugs have minimal effect during the cell cycle in which they are applied, but exhibit maximal lethality during the subsequent cell cycle, even if the drugs have been removed after exposure in the initial cycle [3], [7], [35]–[37]. How this phenomenon works remains uncertain, but the leading hypothesis is that a stockpile of some factor essential to a successful invasion of fresh host cells for the second cell cycle must be accumulated during the first cell cycle, when the drugs take effect [14]. Since actinonin would theoretically be an inhibitor of post-translational modification, we decided to trial its activity over two asexual red blood cell cycles to test for delayed death. Intriguingly, the IC_50_ of 2.7 (±0.15) µm for 96 hours (two cycles) shows only a modest decrease in comparison with the IC_50_ of 4.9 (±1.05) µm over 48 hours (one cycle), suggesting that actinonin does not cause classic delayed death.

Since the putative post-translational inhibitor actinonin was not inducing the anticipated delayed death response, we decided to explore the effect of this inhibitor on turnover of *Pf*Ycf93. Exposure to 20 µM actinonin prevented the degradation of *Pf*Ycf93 that normally occurs during the conversion from schizonts to merozoites then rings between 40 and 56 hours after invasion in the asexual erythrocyte life cycle (Fig. 2A). However, interpretation of the impact of actinonin on turnover of *Pf*Ycf93 is complicated by the fact that this drug also impairs progression though the cell cycle. We exposed parasites with red fluorescent apicoplasts, green fluorescent mitochondria [16] and blue fluorescent DNA to 20 µm actinon and undertook live cell imaging to observe the effect of the drug on organelle development and schizogony (Fig. 2B). Under actinonin drug pressure, apicoplast development and parasite progression through schizogony are both blocked (Fig. 2B). Actinonin treatment of parasites reveals drastic retardation of apicoplast development. At the late trophozoite stage the apicoplast is normally highly branched forming a network around the nuclei [16], but actinonin-treated apicoplasts failed to develop and remained small and rounded, like they are in ring stages (Fig. 2B). Intriguingly, there is virtually no discernible impact of actinonin on the division of mitochondria and nuclei, which branch and divide respectively as normal (Fig. 2B and 2C). There is, however, a marked impact of actinonin treatment on merozoite development; a marker for the parasitophorous vacuole showed drastic defects in membrane structure at the late schizont stage resulting in a failure to undergo cytokinesis (Fig. 2C). We conclude that actinonin blocks apicoplast growth and division, including the turnover of *Pf*Ycf93, likely through inhibition of *Pf*PDF in the apicoplast, though that remains to formally proven.

Immediate death and specific inhibition of apicoplast development in *P. falciparum* (and *P. berghei*) also occurs with exposure to the drug fosmidomycin [38], which inhibits the production of isopentenyl diphosphate [39], an essential product of apicoplasts in the red blood cell stage of the parasite life cycle [40]. We examined the impact of fosmidomycin on *Pf*Ycf93 turnover and observed the same effect as actinonin - abrogation of turnover (Fig. 2B). Similarly, fosmidomycin also blocked apicoplast growth, perturbed schizogony and the appropriate segmentation of the parasitophorous vacuole (Fig. 2C). The remarkable similarity of the drug response phenotypes for actinonin and fosmidomycin perhaps suggests that actinonin perturbs synthesis of isopentenyl diphosphate, but this remains to be established.

Whether or not actinonin impacts directly on *Pf*Ycf93 turnover is difficult to conclude since impairment of apicoplast development by either actinonin or fosmidomycin might indirectly prevent stage specific turnover simply by blocking stage progression. We attempted to determine the actual N-terminus of *Pf*Ycf93 with and without actinonin by immunoprecipitating the protein and N-terminal sequencing by Edman degradation to explore any N-terminal processing by PDF/MAP, but found the N-terminus to be blocked (data not shown). N-terminal blockage for Edman degradation occurs frequently when attempting to sequence proteins [41] and cannot be inferred to result from PDF modification or lack thereof.

### Ycf93 protein family in Apicomplexa

**Figure 3:**
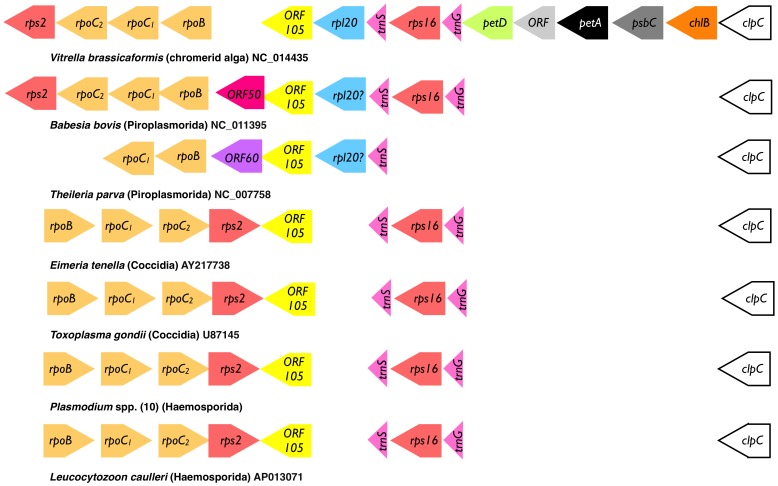
Gene maps (not to scale) for a portion of the IR-B section of the apicoplast genomes of sequenced apicomplexan parasites and the closely related chromerid alga *Vitrella brassicaformis*. Genomic accession numbers and species names are provided above each map. A small open reading frame (orf105/*ycf93*) occurs in syntenic positions in each genome. All the photosynthesis related genes (*petD*, *petA*, *psbC* and *chlB*) from *Vitrella brassicaformis* have disappeared from the apicoplast genomes and reorientation of the *rpo/rps*2 operon has occurred in select species.

**Figure 4:**
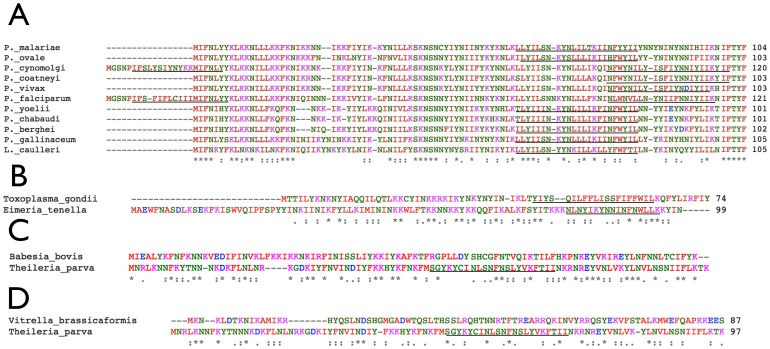
A) Alignment of *Pf*Ycf93 homologues from the 10 sequenced *Plasmodium* spp. and the related bird parasite *Leucocytozoon caulleri* reveals obvious sequence identity and a possible N-terminal extension or an internal initiator methionine in *P. falciparum* and *P. cynomolgi*. Putative transmembrane domains are underlined. B) The *Pf*Ycf93 homologues of the two Coccidia, *Toxoplasma gondii* and *Eimeria tenella*, are well conserved. C) The *Pf*Ycf93 homologues of two Piroplasmorida, *Theileria parva* and *Babesia bovis* also show clear sequence identity. D) Sequence identity between the putative *Vitrella brassicaformis* Ycf93 homologue and the apicomplexan proteins such as *Tp*Ycf93 is far less obvious.

Apicoplast genomes are available for 14 apicomplexan parasites, namely nine species of the Haemosporida genus *Plasmodium* [8], [42], [43], one bird Haemosporida (*Leucocytozoon caulleri* [44]), two Coccidia (*Eimeria tenella* [45] and *Toxoplasma gondii* [46]), and two Piroplasmida (*Babesia bovis* [47], [48] and *Theileria parva* [49]). We examined the IR-B region (or equivalent) of these genomes plus that of the related chromerid algae *Vitrella brassicaformis* [50] and *Chromera velia* [51] for possible orthologues of *ycf93*/*orf105*. Syntenic orthologues of *Pfycf93* with clear sequence identity exist in all sequenced apicoplast genomes of *Plasmodium* spp. plus *Leucocytozoon caulleri* (Figs. 3 and 4). The *Plasmodium* spp. and *L. caulleri* Ycf93 proteins all contain a predicted C-terminal trans membrane domain (Fig. 4A).

Amino acid alignment shows a 16 amino acid N-terminal extension in the *P. falciparum* Ycf93 and a 17 amino acid extension in the Ycf93 orthologue of another primate malaria parasite, *P. cynomolgi* (Fig. 4A). In both cases this extension is predicted to create a second, N-terminal transmembrane domain in addition to the single, predicted C-terminal transmembrane domain. We note that internal methionines in *Pf*Ycf93 and *Pc*Ycf93 occur at the same site as the initiator methionines in the Ycf93 proteins from *L. caulleri* and the other eight malaria parasite species (Fig. 4A), which includes human, bird and rodent infecting species. This internal methionine was initially invoked for *Pf*Ycf93 due to the presence of a transcribed and processed *trnS* gene within the N-terminus of the longer version of *Pf*Ycf93 [10]. A *trnS* gene also occurs in the currently annotated version of *Pc*Ycf93 [43] so we consider it most likely that both *Pf*Ycf93 and *Pc*Ycf93 use this internal methionine for initiation. If *Pf*Ycf93 were in fact initiated from the internal methionine, it would not be a substrate for MAP according to the prediction rules [8]. The apparent mass of *Pf*Ycf93 (slightly greater than predicted) might suggest that the longer version rather than the shorter version is translated, but the addition of a second transmembrane domain in just these two species seems unlikely, and (as mentioned above) confirmation of the N-terminus and of any processing remains to be made.

The apicoplast genomes of the coccidian apicomplexan parasites *Toxoplasma gondii* and *Eimeria tenella* have almost identical architecture and gene content to *Plasmodium* spp. [46] and likely orthologues of *ycf93* occur in these parasites (Fig. 3), though the sequence identity with *Pf*Ycf93 proteins is very low (not shown). Nevertheless, *Tg*Ycf93 and *Et*Ycf93 are similar size proteins, have a predicted C-terminal trans membrane domain, and are well conserved between these two Coccidia (Fig. 4B). Possible *ycf93* orthologues also exist in two members of the Piroplasmorida, namely *Babesia bovis* [47] and *Theileria parva* [49] (Fig. 3). The proteins encoded by these small open reading frames lack much overall sequence identity but are relatively similar within lineage (Fig. 4C). Moreover, a predicted C-terminal trans membrane domain exists in *Tp*Ycf93 (Fig. 4C), and the genes are situated in syntenic position to other members of the family (Fig. 3) so we believe them to be orthologous.

It is also possible that a *ycf93* orthologue exists in the plastids of the related photosynthetic coral symbiont *Vitrella brassicaformis* [50], a chromerid alga related to *Chromera velia* [51]. These algae share a common origin of the plastid with Apicomplexa, but remain photosynthetic [4]. In *V. brassicaformis* good conservation of the gene order in the plastid DNA compared to the apicoplast genome is evident, particularly in comparison with the apicoplast of *B. bovis*, the key difference in this region being the obvious loss of the photosynthesis genes (*petD*, *petA*, *psbC* and *chlB*) in the parasite genome (Fig. 3). A small open reading frame, situated between *rpl20* and *rpoB* in *V. brassicaformis* [4], might be an orthologue of apicoplast *ycf93* (Fig. 3), but it shares only modest sequence identity (Fig. 4D), and, unlike all the orthologues in Apicomplexa, is not predicted to contain any trans membrane domain. Drastic rearrangement of the plastid genome in *C. velia* [4] prevents any identification of putative Ycf93 homologues.

Synteny comparison between the apicoplast genomes and the plastid genome of *V. brassicaformis* in the *ycf93* region indicate that the *rpo/rps2* operon underwent reorientation(s) (Fig. 3). The alga *V. brassicaformis* and the two Piroplasmorida (*B. bovis* and *T. parva*) share the same orientation of this operon, but the Haemosporida (*Plasmodium* and *Leucocytozoon*) and the Coccidia (*Toxoplasma* and *Eimeria*), which share an almost identical architecture [44], have the reverse orientation (Fig. 3). Rearrangements in plastid genomes are common, and it is difficult to interpret a single reorientation in isolation, especially because the Piroplasmorida apicoplast genomes are unusual in being transcribed off a single strand and presumably underwent substantial rearrangements to achieve this.

## Conclusions

We have demonstrated that *orf105/ycf93* from the apicoplast genome of *P. falciparum* encodes a small membrane protein that likely resides in the inner membrane of the apicoplast and undergoes dramatic turnover during the asexual red blood cell life cycle of the parasite. The complete disappearance of this protein after schizogony, and its putative location in the inner apicoplast membrane, might suggest a role in apicoplast biogenesis, but sequence comparison does not give any clues to function. Orthologues of *orf105/ycf93* occur in all apicoplasts, and perhaps the plastid of the related photosynthetic coral symbiont *V. brassicaformis*. In keeping with plastid gene nomenclature convention [11] we elevate the gene from *orf* status to *ycf* (hypothetical chloroplast open reading frame) status as a protein encoded in plastids of multiple organisms, and we occupy the next available number *ycf93*.

## Materials and Methods

### Parasite Culture and Drug Treatment

*Plasmodium falciparum* strains 3D7, D10, mitoYFP/apicoRFP [16], and KAHRP-GFP (Wickham et al., EMBO J 2001) were *in vitro* cultured using standard techniques [52]. We thank the Australian Red Cross for red blood cells. Parasite synchronization was done using sequential sorbitol lysis as previously described [3]. Parasites used were isolated from red blood cells by incubating with 0.15% saponin and 0.1% BSA in PBS at 4°C followed by extensive washing with PBS.

Drug trials were carried out on tightly synchronized parasites using a modified version of the SYBRgreen assay [3], [53]. IC_50_ values were calculated using the four-component inhibition model in Graphpad Prism.

For microscopy and Western analysis of drug treated parasites, inhibitors were added to double synchronised parasites within 4 hours after invasion of a new host cell.

### Peptide Antisera

The peptide NILLKSKNSNNY was selected based on an analysis of the antigenicity and hydrophobicity of the amino acid sequence of *Pf*Ycf93 using VectorNTI (Invitrogen). The peptide was compared to the *P. falciparum* genome and found to be unique for sequences >6 amino acids in length. The peptide was conjugated to *Diphtheria* toxin (Mimotopes) and used to generate polyclonal goat serum (Antibodies Australia). Antibodies were affinity-purified from serum using immobilized peptide as described [54].

### Western Blotting

Protein extraction and Western blotting were done as previously described [54]. For the competition assay, unconjugated *Pf*Ycf93 peptide (Mimotopes) was added to affinity-purified antibodies immediately prior to probing the membrane. Triton X-114 membrane partitioning and subsequent Western blot analysis was carried out as previously described [55]. Affinity purified anti-*Pf*Ycf93, anti-MSP1-19 [56], anti-AMA1 [18], anti-MSP8 [17], and anti-BiP [57] antibodies were used at 5000∶1, 2000∶1, 2000∶1, 1000∶1 and 1000∶1 dilutions, respectively.

### Immunofluorescence Assay

Parasite fixation and labelling was carried out as previously described [27] except for the *Pf*Ycf93 assay in which cell permeabilization and subsequent washing was carried out at 60°C. Affinity purified goat anti-*Pf*Ycf93, rabbit anti-ACP [12] antibodies were use at 250∶1 and 500∶1 respectively. Alexafluor conjugated anti-goat 488 and anti-rabbit 543 (Invitrogen) were used at 1000∶1 dilution. Nuclei were stained with Hoechst 33342. Images were collected using a Leica SP2 confocal microscope and assembled for publication using Adobe Photoshop.

### Bioinformatics

Protein sequences were aligned with ClustalW2 using default parameters [58]. Transmembrane domain predictions were made with THHMM version 2.0 [59].

## References

[1] Tan KR, Magill AJ, Parise ME, Arguin PM (2011) Centers for Disease C, (2011) et al Doxycycline for malaria chemoprophylaxis and treatment: report from the CDC expert meeting on malaria chemoprophylaxis. Am J Trop Med Hyg 84: 517–531. 21460003 10.4269/ajtmh.2011.10-0285 PMC3062442

[2] Krishna S, Staines HM (2012) Non-Antifolate Antibiotics: Clindamycin, Doxycycline, Azithromycin and Fosmidomycin. In: Staines HM, Krisha S, editors. Treatment and Prevention of Malaria: Antimalarial Drug Chemistry, Action and Use: Springer. pp. 141–156.

[3] Goodman CD, Su V, McFadden GI (2007) The effects of anti-bacterials on the malaria parasite *Plasmodium falciparum* . Mol Biochem Parasitol 152: 181–191. 17289168 10.1016/j.molbiopara.2007.01.005

[4] Janouskovec J, Horak A, Obornik M, Lukes J, Keeling PJ (2010) A common red algal origin of the apicomplexan, dinoflagellate, and heterokont plastids. Proc Natl Acad Sci U S A 107: 10949–10954. 20534454 10.1073/pnas.1003335107 PMC2890776

[5] McFadden GI, Waller RF (1997) Plastids in parasites of humans. BioEssays 19: 1033–1040. 9394626 10.1002/bies.950191114

[6] Okamoto N, McFadden GI (2008) The mother of all parasites. Future Microbiol 3: 391–395.

[7] Fichera ME, Roos DS (1997) A plastid organelle as a drug target in apicomplexan parasites. Nature 390: 407–409. 9389481 10.1038/37132

[8] Wilson RJM, Denny PW, Preiser PR, Rangachari K, Roberts K, et al (1996) Complete gene map of the plastid-like DNA of the malaria parasite *Plasmodium falciparum* . J Mol Biol 261: 155–172. 8757284 10.1006/jmbi.1996.0449

[9] El Bakkouri M, Pow A, Mulichak A, Cheung KL, Artz JD, et al (2010) The Clp chaperones and proteases of the human malaria parasite *Plasmodium falciparum* . J Mol Biol 404: 456–477. 20887733 10.1016/j.jmb.2010.09.051

[10] Preiser P, Williamson DH, Wilson RJ (1995) tRNA genes transcribed from the plastid-like DNA of *Plasmodium falciparum* . Nucl Acids Res 23: 4329–4336. 7501453 10.1093/nar/23.21.4329 PMC307387

[11] Hallick RB, Bairoch A (1994) Proposals for the Naming of Chloroplast Genes. III. Nomenclature for Open Reading Frames Encoded in Chloroplast Genomes. Plant Mol Biol Reporter 12: S29–S30.

[12] Waller RF, Cowman AF, Reed MB, McFadden GI (2000) Protein trafficking to the plastid in *Plasmodium falciparum* is via the secretory pathway. EMBO J 19: 1794–1802. 10775264 10.1093/emboj/19.8.1794 PMC302007

[13] Bordier C (1981) Phase separation of integral membrane proteins in Triton X-114 solution. J Biol Chem 256: 1604–1607. 6257680

[14] Ralph SA, van Dooren GG, Waller RF, Crawford MJ, Fraunholz MJ, et al (2004) Metabolic maps and functions of the *Plasmodium falciparum* apicoplast. Nat Rev Microbiol 2: 203–216. 15083156 10.1038/nrmicro843

[15] Zhang YJ, Tian HF, Wen JF (2009) The evolution of YidC/Oxa/Alb3 family in the three domains of life: a phylogenomic analysis. BMC Evol Biol 9: 137. 19534824 10.1186/1471-2148-9-137 PMC2706819

[16] van Dooren GG, Marti M, Tonkin CJ, Stimmler LM, Cowman AF, et al (2005) Development of the endoplasmic reticulum, mitochondrion and apicoplast during the asexual life cycle of *Plasmodium falciparum* . Mol Microbiol 57: 405–419. 15978074 10.1111/j.1365-2958.2005.04699.x

[17] Drew DR, Sanders PR, Crabb BS (2005) *Plasmodium falciparum* merozoite surface protein 8 is a ring-stage membrane protein that localizes to the parasitophorous vacuole of infected erythrocytes. Infect Immun 73: 3912–3922. 15972477 10.1128/IAI.73.7.3912-3922.2005 PMC1168550

[18] Triglia T, Healer J, Caruana SR, Hodder AN, Anders RF, et al (2000) Apical membrane antigen 1 plays a central role in erythrocyte invasion by *Plasmodium* species. Mol Microbiol 38: 706–718. 11115107 10.1046/j.1365-2958.2000.02175.x

[19] Kato Y, Sakamoto W (2013) Plastid protein degradation during leaf development and senescence: Role of proteases and chaperones. Advances in Photosynthesis and Respiration 36: 453–477.

[20] van Dooren GG, Su V, D'Ombrain MC, McFadden GI (2002) Processing of an apicoplast leader sequence in *Plasmodium falciparum* and the identification of a putative leader cleavage enzyme. J Biol Chem 277: 23612–23619. 11976331 10.1074/jbc.M201748200

[21] Ponpuak M, Klemba M, Park M, Gluzman IY, Lamppa GK, et al (2007) A role for falcilysin in transit peptide degradation in the *Plasmodium falciparum* apicoplast. Mol Microbiol 63: 314–334. 17074076 10.1111/j.1365-2958.2006.05443.x

[22] Rathore S, Sinha D, Asad M, Bottcher T, Afrin F, et al (2010) A cyanobacterial serine protease of *Plasmodium falciparum* is targeted to the apicoplast and plays an important role in its growth and development. Mol Microbiol 77: 873–890. 20545854 10.1111/j.1365-2958.2010.07251.x

[23] Lin W, Chan M, Sim TS (2009) Atypical caseinolytic protease homolog from *Plasmodium falciparum* possesses unusual substrate preference and a functional nuclear localization signal. Parasitol Res 105: 1715–1722. 19789896 10.1007/s00436-009-1612-9

[24] Giglione C, Meinnel T (2001) Organellar peptide deformylases: universality of the N-terminal methionine cleavage mechanism. Trends Plant Sci 6: 566–572. 11738381 10.1016/s1360-1385(01)02151-3

[25] Giglione C, Vallon O, Meinnel T (2003) Control of protein life-span by N-terminal methionine excision. Embo J 22: 13–23. 12505980 10.1093/emboj/cdg007 PMC140049

[26] Gardner MJ, Hall N, Fung E, White O, Berriman M, et al (2002) Genome sequence of the human malaria parasite *Plasmodium falciparum* . Nature 419: 498–511. 12368864 10.1038/nature01097 PMC3836256

[27] Tonkin CJ, Van Dooren GG, Spurck TP, Struck NS, Good RT, et al (2004) Localization of organellar proteins in *Plasmodium falciparum* using a novel set of transfection vectors and a new immunofluorescence fixation method. Mol Biochem Parasitol 137: 13–21. 15279947 10.1016/j.molbiopara.2004.05.009

[28] Giglione C, Pierre M, Meinnel T (2000) Peptide deformylase as a target for new generation, broad spectrum antimicrobial agents. Mol Microbiol 36: 1197–1205. 10931273 10.1046/j.1365-2958.2000.01908.x

[29] Giglione C, Meinnel T (2002) The situation on antimicrobial agents and chemotherapy in 2002: Highlights of the 42nd ICAAC. Expert Opin Ther Targets 6: 691–697. 12472381 10.1517/14728222.6.6.691

[30] Madison V, Duca J, Bennett F, Bohanon S, Cooper A, et al (2002) Binding affinities and geometries of various metal ligands in peptide deformylase inhibitors. Biophys Chem 101–102: 239–247. 10.1016/s0301-4622(02)00179-5 12488004

[31] Clements JM, Beckett RP, Brown A, Catlin G, Lobell M, et al (2001) Antibiotic activity and characterization of BB-3497, a novel peptide deformylase inhibitor. Antimicrob Agents Chemother 45: 563–570. 11158755 10.1128/AAC.45.2.563-570.2001 PMC90327

[32] Molteni V, He X, Nabakka J, Yang K, Kreusch A, et al (2004) Identification of novel potent bicyclic peptide deformylase inhibitors. Bioorg Med Chem Lett 14: 1477–1481. 15006385 10.1016/j.bmcl.2004.01.014

[33] Wiesner J, Sanderbrand S, Altincicek B, Beck E, Jomaa H (2001) Seeking new targets for antiparasitic agents. Trends Parasitol 17: 7–8. 10.1016/s1471-4922(00)01735-9 11394347

[34] Bracchi-Ricard V, Nguyen KT, Zhou Y, Rajagopalan PTR, Chakrabarti D, et al (2001) Characterization of an eukaryotic peptide deformylase from *Plasmodium falciparum* . Archives of Biochemistry and Biophysics 396: 162–170. 11747293 10.1006/abbi.2001.2631

[35] Dahl EL, Rosenthal PJ (2007) Multiple antibiotics exert delayed effects against the *Plasmodium falciparum* apicoplast. Antimicrob Agents Chemother 51: 3485–3490. 17698630 10.1128/AAC.00527-07 PMC2043295

[36] Dahl EL, Rosenthal PJ (2008) Apicoplast translation, transcription and genome replication: targets for antimalarial antibiotics. Trends Parasitol 24: 279–284. 18450512 10.1016/j.pt.2008.03.007

[37] Dahl EL, Shock JL, Shenai BR, Gut J, DeRisi JL, et al (2006) Tetracyclines specifically target the apicoplast of the malaria parasite *Plasmodium falciparum* . Antimicrob Agents Chemother 50: 3124–3131. 16940111 10.1128/AAC.00394-06 PMC1563505

[38] Nair SC, Brooks CF, Goodman CD, Strurm A, McFadden GI, et al (2011) Apicoplast isoprenoid precursor synthesis and the molecular basis of fosmidomycin resistance in *Toxoplasma gondii* . J Exp Med 208: 1547–1559. 21690250 10.1084/jem.20110039 PMC3135366

[39] Jomaa H, Wiesner J, Sanderbrand S, Altincicek B, Weidemeyer C, et al (1999) Inhibitors of the nonmevalonate pathway of isoprenoid biosynthesis as antimalarial drugs. Science 285: 1573–1576. 10477522 10.1126/science.285.5433.1573

[40] Yeh E, DeRisi J (2011) Chemical rescue of malaria parasites lacking an apicoplast defines organelle function in blood-stage *Plasmodium falciparum* . PLoS Biology 9: e1001138. 21912516 10.1371/journal.pbio.1001138 PMC3166167

[41] Wellner D, Panneerselvam C, Horecker BL (1990) Sequencing of peptides and proteins with blocked N-terminal amino acids: N-acetylserine or N-acetylthreonine. Proc Natl Acad Sci U S A 87: 1947–1949. 2106685 10.1073/pnas.87.5.1947 PMC53601

[42] Arisue N, Hashimoto T, Mitsui H, Palacpac NM, Kaneko A, et al (2012) The *Plasmodium* apicoplast genome: conserved structure and close relationship of *P. ovale* to rodent malaria parasites. Mol Biol Evol 29: 2095–2099. 22396524 10.1093/molbev/mss082

[43] Sato S, Sesay AK, Holder AA (2013) The unique structure of the apicoplast genome of the rodent malaria parasite *Plasmodium chabaudi chabaudi* . PLoS One 8: e61778. 23613931 10.1371/journal.pone.0061778 PMC3627918

[44] Imura T, Sato S, Sato Y, Sakamoto D, Isobe T, et al. (2013) The apicoplast genome of *Leucocytozoon caulleryi*, a pathogenic apicomplexan parasite of the chicken. Parasitol Res published online 04 December 2013 DOI 10.1007/s00436-013-3712-9. 10.1007/s00436-013-3712-9 PMC3932168 24301182

[45] Cai X, Fuller AL, McDougald LR, Zhu G (2003) Apicoplast genome of the coccidian *Eimeria tenella* . Gene 321: 39–46. 14636990 10.1016/j.gene.2003.08.008

[46] Köhler S, Delwiche CF, Denny PW, Tilney LG, Webster P, et al (1997) A plastid of probable green algal origin in apicomplexan parasites. Science 275: 1485–1488. 9045615 10.1126/science.275.5305.1485

[47] Brayton KA, Lau AO, Herndon DR, Hannick L, Kappmeyer LS, et al (2007) Genome sequence of *Babesia bovis* and comparative analysis of apicomplexan hemoprotozoa. PLoS Pathog 3: 1401–1413. 17953480 10.1371/journal.ppat.0030148 PMC2034396

[48] Lau AO, McElwain TF, Brayton KA, Knowles DP, Roalson EH (2009) *Babesia bovis*: a comprehensive phylogenetic analysis of plastid-encoded genes supports green algal origin of apicoplasts. Exp Parasitol 123: 236–243. 19646439 10.1016/j.exppara.2009.07.007

[49] Gardner MJ, Bishop R, Shah T, de Villiers EP, Carlton JM, et al (2005) Genome sequence of *Theileria parva*, a bovine pathogen that transforms lymphocytes. Science 309: 134–137. 15994558 10.1126/science.1110439

[50] Obornik M, Modry D, Lukes M, Cernotikova-Stribrna E, Cihlar J, et al (2012) Morphology, ultrastructure and life cycle of *Vitrella brassicaformis n. sp., n. gen.*, a novel chromerid from the Great Barrier Reef. Protist 163: 306–323. 22055836 10.1016/j.protis.2011.09.001

[51] Moore RB, Obornik M, Janouskovec J, Chrudimsky T, Vancova M, et al (2008) A photosynthetic alveolate closely related to apicomplexan parasites. Nature 451: 959–963. 18288187 10.1038/nature06635

[52] Trager W, Jensen JB (1976) Human malaria parasites in continuous culture. Science 193: 673–675. 781840 10.1126/science.781840

[53] Smilkstein M, Sriwilaijaroen N, Kelly JX, Wilairat P, Riscoe M (2004) Simple and inexpensive fluorescence-based technique for high-throughput antimalarial drug screening. Antimicrob Agents Chemother 48: 1803–1806. 15105138 10.1128/AAC.48.5.1803-1806.2004 PMC400546

[54] Mullin KA, Lim L, Ralph SA, Spurck TP, Handman E, et al (2006) Membrane transporters in the relict plastid of malaria parasites. Proc Natl Acad Sci U S A 103: 9572–9577. 16760253 10.1073/pnas.0602293103 PMC1480448

[55] Kalanon M, Tonkin CJ, McFadden GI (2009) Characterization of two putative protein translocation components in the apicoplast of *Plasmodium falciparum* . Eukaryot Cell 8: 1146–1154. 19502580 10.1128/EC.00061-09 PMC2725556

[56] Boyle MJ, Richards JS, Gilson PR, Chai W, Beeson JG (2010) Interactions with heparin-like molecules during erythrocyte invasion by *Plasmodium falciparum* merozoites. Blood 115: 4559–4568. 20220119 10.1182/blood-2009-09-243725

[57] Kumar N, Koski G, Harada M, Aikawa M, Zheng H (1991) Induction and localization of *Plasmodium falciparum* stress proteins related to the heat shock protein 70 family. Mol Biochem Parasitol 48: 47–58. 1779989 10.1016/0166-6851(91)90163-z

[58] Larkin MA, Blackshields G, Brown NP, Chenna R, McGettigan PA, et al (2007) Clustal W and Clustal X version 2.0. Bioinformatics 23: 2947–2948. 17846036 10.1093/bioinformatics/btm404

[59] Krogh A, Larsson B, von Heijne G, Sonnhammer EL (2001) Predicting transmembrane protein topology with a hidden Markov model: application to complete genomes. J Mol Biol 305: 567–580. 11152613 10.1006/jmbi.2000.4315

